# Antibacterial Potential of *Jatropha curcas* Synthesized Silver Nanoparticles against Food Borne Pathogens

**DOI:** 10.3389/fmicb.2016.01748

**Published:** 2016-11-08

**Authors:** Nitin Chauhan, Amit K. Tyagi, Pushpendar Kumar, Anushree Malik

**Affiliations:** ^1^Applied Microbiology Laboratory, Centre for Rural Development and Technology, Indian Institute of Technology DelhiNew Delhi, India; ^2^Cytokine Research Laboratory, Department of Experimental Therapeutics, The University of Texas MD Anderson Cancer CentreHouston, TX, USA

**Keywords:** *Jatropha curcas*, silver nanoparticles, antibacterial, *Listeria monocytogenes*

## Abstract

The aqueous leaf extract of *Jatropha curcas* was used for the synthesis of silver nanoparticles (Jc-AgNps) which were further evaluated for its antibacterial potential against food borne pathogens. *J. curcas* leaf extract could synthesize stable silver nanoparticles (Zeta potential: -23.4 mV) with absorption band at 430 nm. Fourier transform infrared spectroscopy indicated various biological compounds responsible for capping and stabilizing Jc-AgNps in suspension, while the presence of silver was authenticated by scanning electron microscopy (SEM) equipped with energy-dispersive X-ray. Jc-AgNps were confirmed to be uniform in shape, size and behavior through dynamic light scattering, transmission electron microscopy (TEM), X-ray diffraction, SEM, and atomic force microscopy (AFM) analysis. To investigate the antibacterial activity, disk diffusion and microplate dilution assays were performed and zone of inhibition (ZOI) as well as minimum inhibitory/bactericidal concentrations (MIC/MBCs) were evaluated against selected bacterial strains. Overall results showed that *Escherichia coli* (ZOI: 23 mm, MBC: 0.010 mg/ml) was the most sensitive organism, whereas *Staphylococcus aureus* (ZOI: 14.66 mm, MBC: 0.041 mg/ml) and *Salmonella enterica* (ZOI: 16.66 mm, MBC: 0.041 mg/ml) were the least sensitive against Jc-AgNps. The detailed microscopic investigations using SEM, TEM, and AFM were performed to understand the antibacterial impacts of Jc-AgNps against *Listeria monocytogenes*. SEM and TEM analysis showed the clear deformation and disintegration of treated *L. monocytogenes* cells, whereas AFM established a decrease in the height and cell surface roughness (root mean square value) in the treated *L. monocytogenes*.

## Introduction

Nanotechnology has already been applied in various fields such as electronics (Daniel and Astruc, 2004), optics (Porel et al., 2005), catalysis (Tsunoyama et al., 2004; Behera and Ram, 2013), medicine (Singh et al., 2008), and environment remediation (Afkhami and Moosavi, 2010; Abbassi et al., 2013). However, its use in the food industry, especially as antimicrobial agent for food borne pathogens, is just beginning to develop. In food industries, nanoparticles with proven antimicrobial efficacy against food spoiling microbes can find valuable application. Among the studied nanoparticles, silver being the efficient antimicrobial agent, is attracting the researchers to design and develop the ways for its proper delivery and efficient action (Yeo and Jeong, 2003; Samuel and Guggenbichler, 2004). Green synthesis of silver nanoparticles (AgNps) is considered advantageous over chemical or physical methods due to simple, cost effective, and considerably safe production methodology (Gurunathan et al., 2013; Hebbalalu et al., 2013; de Faria et al., 2014). Microbes or plants can be employed for the green synthesis. However, due to various hurdles associated with the maintenance of pure microbial cultures, plants are considered as the best candidates for synthesis of AgNps (Ahmed et al., 2016).

Recent studies have highlighted the antibacterial activity of plant based AgNps against broad range of Gram-positive and Gram-negative bacteria (Jayaprakash et al., 2014; Salem et al., 2014; Muthukrishnan et al., 2015; Rajakannu et al., 2015; Sre et al., 2015). Although the actual mechanism of AgNps functioning is not yet clear, researchers believe that AgNps interact with thiol group of bacterial proteins (Lok et al., 2006) and phosphorous moieties of DNA (Rai et al., 2009) to inactivate the bacterial cell system. It was also reported that nano-silver is non-toxic to humans at low doses (Wen et al., 2007; Tankhiwale and Bajpai, 2009). Thereafter, few attempts were made to develop AgNps coated systems, especially for food preservation against lethal food borne pathogens. Tankhiwale and Bajpai (2009) used the graft copolymerization of vinyl monomers onto cellulose based filter paper followed by entrapment of AgNps. The developed nano-silver based food packaging was found to be effective against *Escherichia coli*. Similarly, AgNps incorporated onto hydroxypropyl methylcellulose (HPMC) matrix were found to show antibacterial activity against *E. coli* and *Staphylococcus aureus* (De Moura et al., 2012). However, these materials have employed chemical/physical methods for synthesis and stabilization of AgNps. Therefore, green routes for AgNps synthesis and subsequent development of such systems is the need of the hour.

*Jatropha curcas* (Euphorbiaceae) is widely cultivated for commercial scale bio diesel production from its seeds (Berchmans and Hirata, 2008; Tapanes et al., 2008). *J. curcas* latex has been reported for the medicinal uses like wound healing and blood coagulant activity (Osoniyi and Onajobi, 2003). The leaf extract of *J. curcas* was also reported for insecticidal and antimicrobial properties (Kalimuthu et al., 2010; Verma et al., 2013; Chauhan et al., 2015). Moreover, *J. curcas* leaf extract has recently been used for the synthesis of AgNps, however, no antimicrobial properties were investigated (Rajasekharreddy et al., 2010). Therefore, in the present study, an attempt was made to synthesize the AgNps from aqueous leaf extract of *J. curcas* and to study the antimicrobial activity of *J. curcas* leaf synthesized silver nanoparticles (Jc-AgNps) against various food borne pathogens including Gram-positive (*Bacillus cereus*, *Staphylococcus aureus*, *Listeria monocytogenes*) and Gram-negative bacteria (*Escherichia coli*, *Pseudomonas aeruginosa*, *Salmonella enterica*). Since few studies have highlighted the interaction of AgNps with bacterial cells and subsequent cell damage, (Sondi and Salopek-Sondi, 2004; Morones et al., 2005; Kim et al., 2011; Das et al., 2015), these aspects were investigated in the current study. A detailed microscopic analysis of food borne pathogen *L. monocytogenes* was carried out using scanning electron microscopy (SEM), transmission electron microscopy (TEM), and atomic force microscopy (AFM) techniques to visualize the impact of Jc-AgNps on cell morphology and ultrastructure. To the best of author’s knowledge, this is the first report to visualize the effect of Jc-AgNps on the morphology of *L. monocytogenes* cells through various microscopic analysis, thereby strengthening the approach of “AgNps green synthesis” for designing safe and active materials for food preservation.

## Materials and Methods

### Chemicals

Silver nitrate used in the study was of analytical grade purchased from Merck, India. Mueller–Hinton broth and Mueller–Hinton agar for antimicrobial activity were purchased from Hi-Media, Mumbai, India. The aqueous solutions were prepared using ultra filtered water (resistivity: 18.2 M Ω-cm).

### Plant Sample Collection and Extract Preparation

Disease-free, healthy leaves of *Jatropa curcas* were collected during the month of May in 2012 from Micro model complex, Indian Institute of Technology Delhi, New Delhi, India. The collected leaves were washed thoroughly with tap water and then rinsed with distilled water until no foreign material remained. Ten grams of freshly chopped leaves of *J. curcas* were mixed with 100 ml of Milli Q water in a 500 ml beaker and warmed in water bath at 60°C for 10 min. The extract was filtered with filter paper (Whatman’s filter paper no. 1). The filtered leaf extract was kept at 4°C till the synthesis of AgNps.

### Bio-Synthesis of Silver Nanoparticles from the Aqueous Leaf Extract of *J. curcas*

Five milliliters of leaf extract was added into 45 ml of 0.002 M AgNO_3_ solution for the reduction of silver ions in a 100 ml Erlenmeyer flask at room temperature. The reaction mixture was kept in dark room condition until the color change was initiated. The brownish–gray color of solution indicated the formation of the silver nanoparticles (Jc-AgNPs). The bio-reduction of the silver ions was monitored through the UV-visible spectroscopy (300–700 nm) of the solutions. The water-suspended nanoparticles were frozen (-40°C) for 24 h prior to lyophilisation. The frozen samples were lyophilised (Allied frost FD-3) under -80°C for 2 days. The structure and composition of lyophilised Jc-AgNPs were evaluated by SEM, TEM, AFM, energy-dispersive X-ray (EDX), and fourier transform infra-red spectroscopy (FTIR) and X-ray diffraction (XRD).

### Characterization of Jc-AgNPs

#### UV-vis Spectra Analysis

The reduction of silver ions was observed by measuring the UV-vis spectrum with Perkin Elmer Preclsley, Lambda 35 Spectrophotometer operated at a resolution of ±1 nm at different time intervals (Roy et al., 2014). UV-vis spectroscopic analysis was performed by continuous scanning from 300 to 700 nm and 0.002 M AgNO_3_ solution was used for the baseline correction.

#### Quantification of Jc-AgNps

The sample of Jc-AgNps (1 mg/ml) was digested in microwave digestion (160 ± 4°C within 10 min and 165–170°C for 10 min for reaction) and analyzed further for quantification of AgNps concentration using Microwave plasma-atomic emission spectrometry (Agilent 4200 MP-AES) (Gola et al., 2016). The conditions for MP-AES were maintained as (i) Nebulizer flow: 0.65 L/min (ii) Stabilization time: 15 s (iii) Read time: 3 s (iv) Pump speed: 15 rpm, (v) Background correction: Auto (v) Analyte (wavelength): Ag (328.06 nm).

#### SEM and EDX Analysis

The lyophilised Jc-AgNPs were characterized for nanoparticles shape with a scanning electron microscope (ZEISS EVO 50). EDX analysis was conducted with the same instrument to confirm the presence and elemental composition of the synthesized sample as described before (Forough and Farhadi, 2010).

#### TEM Analysis

The drop of purified aqueous Jc-AgNps sample was loaded on carbon-coated copper grid and it was allowed to dry at room temperature for 4 h. The TEM micrograph images were recorded on Philips transmission electron microscope (CM-10) on carbon coated copper grids with an accelerating voltage of 70 kV (Balaji et al., 2009). The clear microscopic views were observed and documented at different range of magnifications.

#### AFM Analysis

Further to analyze the particle size and characterization of these nanoparticles, AFM was used (Dwivedi et al., 2011; Prathna et al., 2011). The microscopic images were recorded with silicon cantilever with force constant 0.22–0.77 N/m, tip height 10–12 nm in the contact mode. The Jc-AgNps were studied in three dimensional views with various other structural parameters.

#### FTIR Analysis

To identify the biomolecules present in the Jc-AgNPs after the synthesis of the AgNps, FTIR spectra of lyophilised Jc-AgNPs powder were analyzed by FTIR spectroscopy (Perkin Elmer One spectrum) (Prathna et al., 2011). The FTIR analysis was performed with KBr pellets. The FTIR was recorded in a diffuse reflection mode at a resolution of 4 cm^-1^. The various modes of vibrations were identified to determine the different functional groups present in the Jc-AgNPs.

#### X-ray Diffraction (XRD) Measurement

Crystalline metallic silver was also examined by XRD using X’ Pert PRO Philips Analytical-PW 3040/60 X ray diffractometer with a CuKα-radiation monochromatic filter in the range 35–80°.

#### Zeta Potential (ζ-potential) and Dynamic Light Scattering (DLS) Measurements

The particle size distribution and ζ-potential of the Jc-AgNps solutions were measured using a particle analyzer (Nano ZS90 Zetasizer, Malvern Instruments, UK) equipped with a He-Ne laser (633 nm, 5 mW) (Edison and Sethuraman, 2012). The numbers of measurements were optimized automatically by the software.

#### Bacterial Culture Preparation

Bacterial strain such as *Escherichia coli* (ATCC 25922), *Pseudomonas aeruginosa* (ATCC 9027), *Salmonella enterica* Serovar Enteritidis (ISO 155), *Bacillus cereus* (ATCC 11966), *Staphylococcus aureus* (SR 231), were collected from the Central Microbial Culture Facility, Department of Biotechnology and Biochemical Engineering, Indian Institute of Technology Delhi, New Delhi, India, while *Listeria monocytogenes* (SCOTT-A) was obtained from the Strain Collection of Dipartimento di Scienze degli Alimenti, University of Bologna, Italy. The collected strains were grown in Mueller-Hinton broth (MHB) medium for 24 h in an orbital shaking incubator (Scigenics India Pvt. Ltd., India) at 180 rpm and 30°C. Cells were harvested by centrifugation (5000 × *g*, 10 min), suspended in saline and used immediately.

### Antimicrobial Assays

#### Disk Diffusion Method

As described by Tyagi and Malik (2011), the aliquot of 100 μl from each cell suspension containing approximately 10^5^ cfu/ml was spread over the surface of MHA plate and allowed to dry. The sterile paper disks (diameter 6 mm, Sigma–Aldrich Inc., India) were placed on agar plates and 10-30 μl of Jc-AgNps solution (0.5 mg/ml) was poured on each disk. The plates were then sealed with parafilm to prevent any unwanted contamination. All the plates were incubated at 30°C for 24 h and the diameter of the resulting inhibition zone in each plate was measured. All tests were performed in triplicate.

#### Determination of MIC and MBC by Microplate Dilution Method

Minimum Inhibitory Concentration (MIC) and Minimum Bactericidal Concentration (MBC) of synthesized Jc-AgNps were determined by microplate dilution method. Different concentrations of Jc-AgNps were prepared using ultrapure water and added to the microtiter wells to obtain the final concentrations of 0.005, 0.010, 0.020, 0.041, 0.083, 0.166, and 0.333 mg/ml. The components such as MHB media (100 μl); 1×10^5^ bacterial culture (100 μl); different concentrations (0.005–0.333 mg/ml) of Jc-AgNps (100 μl) were added in each well of 96-well microplate (Mariselvam et al., 2014; Yehia and Al-Sheikh, 2014). The control sample contained every component, i.e., bacterial cells in saline and sterile distilled water without nanoparticles. Further, the microplates were incubated at 30°C. The visual observation of the microplates for bacterial growth was performed after 24 and 48 h of the incubation. The MIC values were identified as the minimum concentration at which no visible bacterial growth was recorded. For MBC, an aliquot of 50 μl from all the wells showing no visible bacterial growth were applied on MHA plates and incubated at 30°C for 24 h. The MBC were observed as the lowest concentration that completely inhibited the bacteria.

#### Characterization of Bacterial Cells

The bacterial cells of *L. monocytogenes and S. enterica* were incubated for 14 h in MHB medium at 30°C and 180 rpm. *L. monocytogenes* cell suspension was divided into two parts. In first part (treated), the MIC concentrations, (0.010 mg/ml) was added while the second part was left untreated (control). The suspensions were incubated for 8 h at 30°C. Similarly, *S. enterica* was treated at MIC and ½ MIC level. All the treated and untreated cells were harvested by centrifugation and were prefixed with a 2.5% glutaraldehyde solution overnight at 4°C. After this, the cells were again harvested by centrifugation and washed three times with 0.1 M sodium phosphate buffer solution (pH 7.2). Now each resuspension was serially dehydrated with 25, 50, 75, 90, and 100% ethanol, respectively. Then, cells were dried at “critical point.” For SEM, a thin film of cells was smeared on a silver stub. The samples were gold-covered by cathodic spraying (Polaron gold). Finally, morphology of the *L. monocytogenes* and *S. enterica* was observed on a scanning electronic microscope (ZEISS EVO 50). The SEM observation was done under the following analytical condition: EHT = 20 kv, WD = 10 mm, Signal A = SE_1_. For TEM, the pellet was post fixed in 1% osmium tetraoxide for 30 min, washed with phosphate buffer solution (pH 7.2), serially dehydrated in ethanol and embedded in Epon-Araldite resin for making the blocks of the cells pellet. Ultra thin (50–100 nm) sections of the cells were stained with uranyl acetate with lead citrate and observed under a Philips transmission electron microscope (CM-10) at 100 eV and direct magnification of 50000×. For AFM mounting of bacterial cell (*L. monocytogenes*), glass substrates were employed. Ten microliters of untreated and treated bacterial cells suspension was mounted on a glass substrate. After air-drying the cells were imaged with AFM in contact mode.

### Statistical Analysis

All the experiments were done in triplicate and the data presented here represents the mean of three replicates. Data related to the zone of inhibition (ZOI) were subjected to analysis of variance (one-way ANOVA) in Duncan multiple range test using SPSS (2008; version 17.5) statistical software. The differences with *p* < 0.05 were considered significant.

## Results

The AgNps produced using aqueous leaf extract of *J. curcas* were characterized and its antibacterial property was evaluated against selected bacterial strains followed by the detailed microscopic characterization of the treated bacterial cells.

### Physical Characterization of Jc-AgNps

UV-vis spectra confirmed the synthesis of Jc-AgNps as evident from the peak at 430 nm (**Supplementary Figure S1B**). The change in color from yellowish to deep brown with time is due to excitation in surface plasmon resonance (**Supplementary Figure S1A**). There is no color change in *J. curcas* leaf extract and AgNO_3_ solution alone, confirming that components from leaf extract actually reduced the metallic silver into AgNps. The pH of the Jc-AgNp solution was observed to be 6.8 after 72 h. The lyophilized sample (1 mg/ml, Jc-AgNp) was diluted (200 times) for accurate measurement before analysis through MP-AES. The calibration curve was prepared (**Supplementary Figure S2A**) using three different concentrations of silver (2, 6, and 8 ppm), further the analysis of the sample (Jc-AgNp) was performed. The result obtained from MP-AES analysis indicated that the stock of 1 mg/ml of Jc-AgNps has the concentration of 550 ppm of AgNps (**Supplementary Figure S2B**), hence the dilutions of 0.005, 0.010, 0.020, 0.041, 0.083, 0.166, and 0.333 mg/ml used in the antibacterial experiments contained 2.75, 5.5, 11, 22.55, 45.65, 91.3, 183.15 ppm of AgNps, respectively. The analysis such as Zeta potential and DLS were performed to determine the stability of Jc-AgNps in suspension. A negative zeta potential of Jc-AgNps (-23.4 mV) proved the high stability of AgNps in colloidal solution (**Supplementary Figure S3B**). DLS analysis showed that the mean diameter of the Jc-AgNps is 43.67 nm (**Supplementary Figure S3A**) proving the nano-size of synthesized Jc-AgNps.

To assess whether these nanoparticles are crystalline in nature, XRD analysis was conducted. As shown in **Supplementary Figure S3C**, the diffraction peaks at 2𝜃 = 38.28°, 46.25°, and 77.62° were assigned to diffraction signals (111), (200), and (311) of face centered cubic (fcc) structures of Jc-AgNps as per the Joint Committee on Powder Diffraction Standards (JCPDS) file no: ICDD-PDF2, PA, USA, 2007. Next, to identify Jc-AgNps associated compounds, FTIR spectra was recorded. The FTIR spectra of Jc-AgNps exhibited prominent peaks at 3321, 1608, 1343, 1043, and 774 cm^-1^ (**Supplementary Figure S3D**). The peaks 1608 cm^-1^ represented the involvement of C-N in plane vibrations of amino-acids and 1020–1220 cm^-1^ represented the involvement of C-N in plane vibrations of aliphatic amines. The presence of peaks due to O-H stretching (around 3321 cm^-1^), C-C and C=O stretching (1343 cm^-1^), O-H stretching (1043 cm^-1^), and C-H alkenes (774 cm^-1^) was also observed highlighting the presence of various biological compounds for stabilization of AgNps in *J. curcas* extract.

### Microscopic Evaluation of Jc-AgNps

**FIGURE 1:**
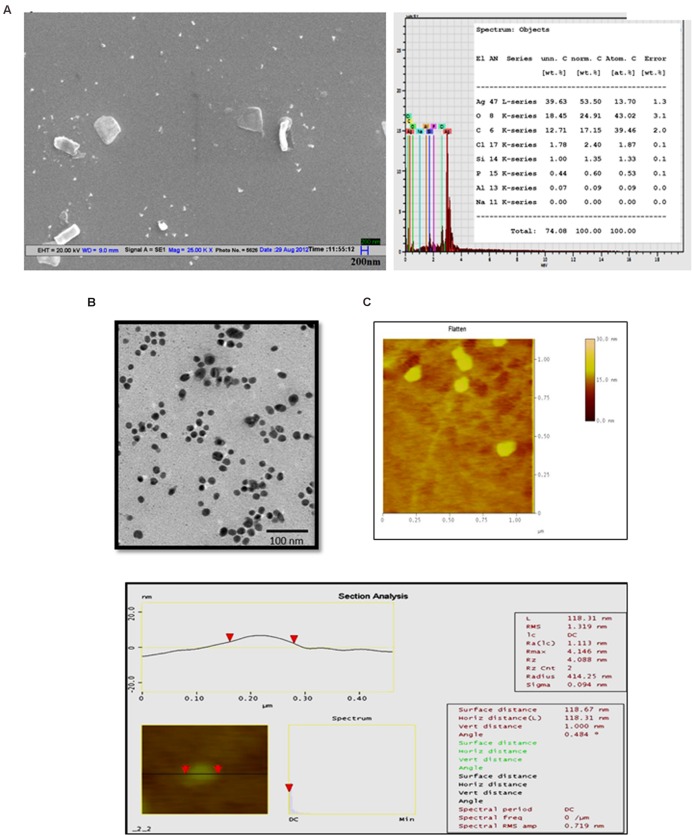
**Microscopic analysis of green synthesized Jc-AgNps. (A)** Left: SEM image of Jc-AgNps showing variation in sizes (50–100 nm); Right: energy-dispersive X-ray (EDX) patterns of Jc-AgNps showing strong signals of silver (20 kV), **(B)** Transmission electron microscopic images of Jc-AgNPs. Images were taken at 200 kV on a carbon-coated Cu grid, **(C)** AFM image showing root mean square along with major statistical parameters of Jc-AgNps.

The lyophilized Jc-AgNps were analyzed for shape, size, and elemental composition under SEM equipped with energy dispersive X-ray spectroscopy. The diameter of nanoparticles was found to be in range of 50–100 nm, although some clumps were also observed (**Figure 1A**, left panel). The EDX analysis of Jc-AgNps showed strong signals (20 kV) of silver, whereas weaker signals were recorded for other elements including oxygen, carbon, chloride, and silicon from synthesized sample (**Figure 1A**, right panel). The TEM analysis clearly showed variation in particle shape and size (20–50 nm; **Figure 1B**). While majority of the Jc-AgNps were spherical, some showed irregular shapes and edges. AFM was conducted to understand the surface properties of synthesized nanoparticles. The corresponding section analysis of Jc-AgNPs is shown in **Figure 1C**. The RMS value corresponds to the roughness of the sample and its value was observed to be 1.31 nm. The analysis proved that the synthesized Jc-AgNps were ∼100 nm correlating with the results of TEM or SEM analysis.

### Antimicrobial Activity of Jc-AgNps

#### Zone of Inhibition

**FIGURE 2:**
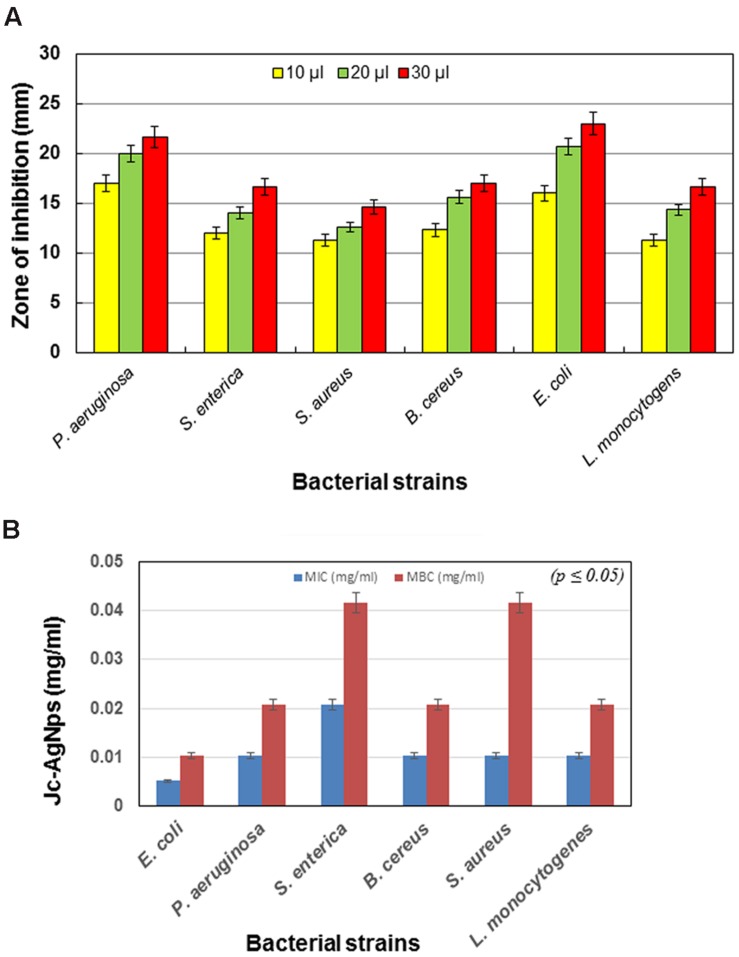
**Antibacterial activity of green synthesized Jc-AgNps. (A)** Zone of inhibition (mm) due to different volumes (10, 20, and 30 μl/disk, concentration: 0.5 mg/ml) of Jc-AgNps against bacterial strains, **(B)** Minimum Inhibitory/Bactericidal Concentrations (MIC/MBCs) for different bacterial strains.

The antimicrobial activity of Jc-AgNps measured in terms of ZOI is shown in **Figure 2A**. It was observed that the ZOI increased in dose-dependent manner and followed the same trend with respect to different bacterial strains. The highest inhibitory zone (23 mm) was observed in *E. coli* at 30 μl volume, whereas the lowest inhibitory zone (14.6 mm) was found with *S. aureus*. However, *P. aeruginosa* was found to be more sensitive than *E. coli* at 10 μl volume. Based on the overall results obtained from the ZOI data, the pattern of sensitivity was observed in the order as *E. coli* > *P. aeruginosa* > *B. cereus* > *S. enterica* = *L. monocytogenes* > *S. aureus.*

#### MIC and MBC of Jc-AgNps

The MIC and MBC values of AgNps against various Gram-positive (*B. cereus*, *S. aureus*, *L. monocytogenes*) and Gram-negative bacteria (*E. coli*, *P. aeruginosa*, *S. enterica*) is shown in **Figure 2B**. For Gram-negative bacteria, the MIC varied from 0.005 to 0.020 mg/ml, whereas for Gram-positive bacteria, it was found to be 0.010 mg/ml. Similarly, for Gram-negative bacteria, the value of MBC varied between 0.010 and 0.041 mg/ml and for Gram-positive bacteria, it was observed to be 0.020 and 0.041 mg/ml. The highest value of MIC (0.020 mg/ml) was observed with *S. enterica*, whereas the lowest MIC (0.005 mg/ml) was observed with *E. coli*. Similarly, for MBC, the highest value (0.041 mg/ml) was observed with *S. aureus* and *S. enterica*, whereas the lowest value (0.010 mg/ml) was observed with *E. coli*.

#### Effect of Jc-AgNps on Bacterial Cell Morphology and Ultrastructure

To gain insight into the interaction of Jc-AgNps with the bacterial cells leading to the cell death, detailed microscopic characterization was conducted. Gram-positive *L. monocytogenes*, which is the most virulent food borne pathogen, causes meningitis and is the leading cause of death among food borne pathogens (Donnelly, 2001), was selected. Gram-negative *S. enterica* was also studied for comparative evaluation.

**FIGURE 3:**
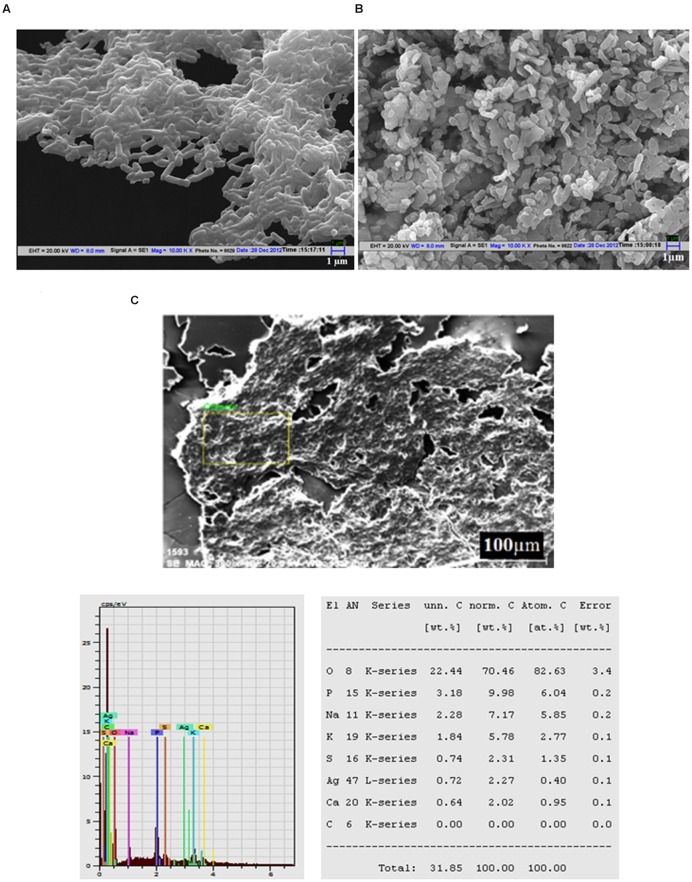
**Scanning electron microscopic (SEM) analysis. (A)** SEM micrograph of untreated *Listeria monocytogenes* cells showing normal and smooth surface, **(B)** SEM micrograph of Jc-AgNps treated *L. monocytogenes* cells showing shrinkage and deformation, **(C)** SEM-EDX analysis of treated *L. monocytogenes* cells (silver: 0.72 wt.%).

Scanning electron microscopy analysis of untreated cells showed the normal and smooth surfaces of *L. monocytogenes* (**Figure 3A**), whereas the cells treated with Jc-AgNps at MIC level showed shrinkage and deformation (**Figure 3B**). The micrograph also depicts scattered cell debris around the cells. The SEM equipped EDX analysis confirmed the presence of silver (0.72 wt. %) in the treated cells of *L. monocytogenes* (**Figure 3C**) establishing the role of AgNps for cell damages. Similarly, the treated cells of *S. enterica* showed dose dependent morphological deformity (at MIC and ½ MIC) in comparison with well organized and intact morphology of untreated cells under SEM examination (**Supplementary Figure S4**).

**FIGURE 4:**
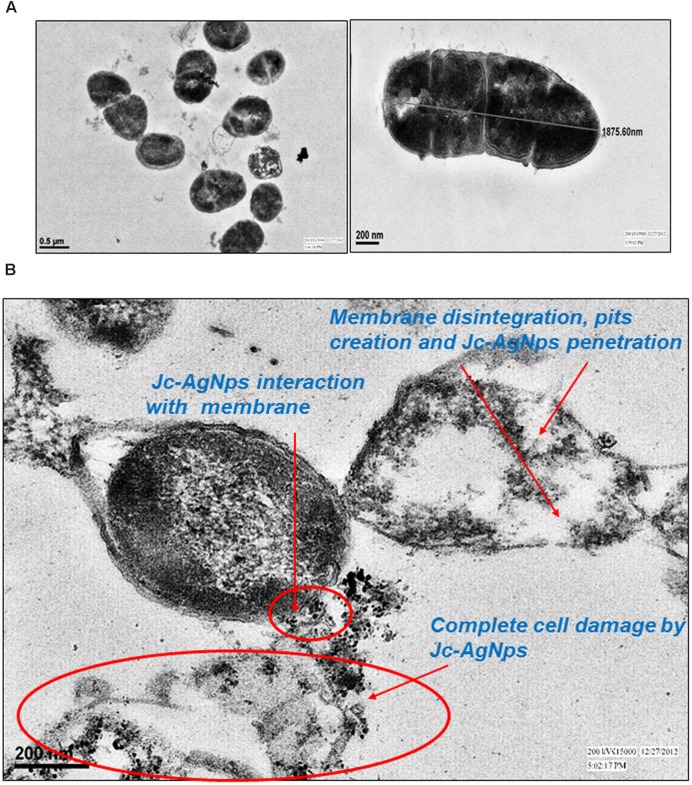
**Transmission electron microscopic (TEM) examination. (A)** TEM micrograph of untreated *L. monocytogenes* cells showing normal membrane and intact cell, **(B)** TEM micrograph of Jc-AgNps treated *L. monocytogenes* cells showing biocidal actions including; Jc-AgNps adherence and interaction with membrane; Membrane disintegration, pits creation and complete cell damage.

Next, TEM analysis was performed to study the ultrastructure of treated and untreated *L. monocytogenes* cells. The untreated cells showed a well-defined cell membrane with normal intact cells and dense cytoplasm (**Figure 4A**). After exposure to Jc-AgNps at MIC level, the cell membrane was observed to be disrupted and disintegrated (**Figure 4B**). The cell was ruptured and showed disorganized cytoplasm with noticeable AgNps at various sites confirming the role of silver for these damages as suggested through SEM-EDX analysis.

**FIGURE 5:**
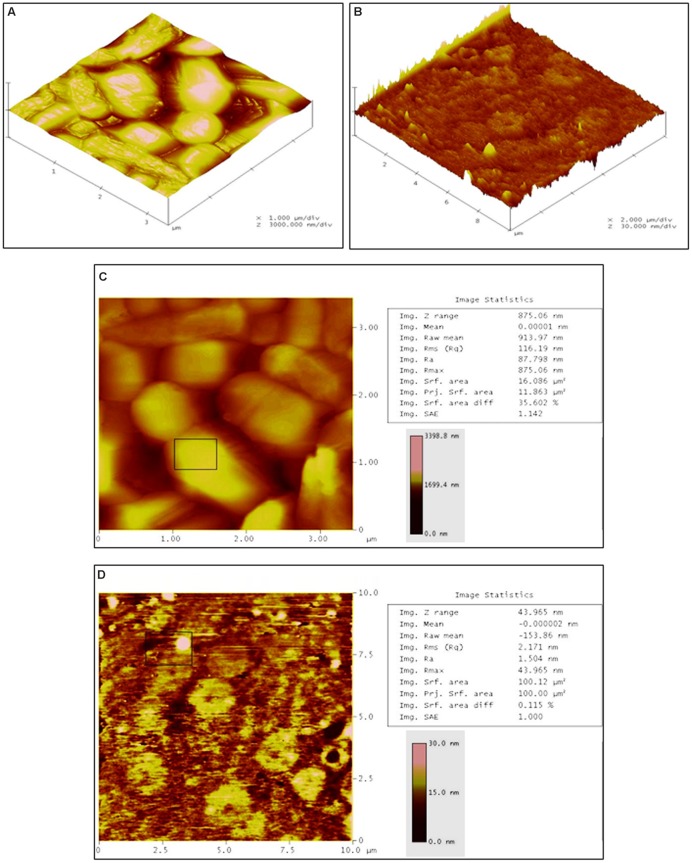
**Atomic force microscopic analysis. (A)** Three dimensional section of untreated *L. monocytogenes* cells showing normal and smooth cell surfaces, **(B)** Three dimensional section of Jc-AgNps treated *L. monocytogenes* cells showing clear cell damage, **(C)** Image statistics of untreated *L. monocytogenes* cells (RMS: 116.19 nm, height: 1699 nm), **(D)** Image statistics of treated *L. monocytogenes* cells (RMS: 2.17 nm, height:15 nm).

To determine the three dimensional morphological changes along with roughness of cell surfaces in treated and untreated cells, AFM analysis was carried out. As compared to the untreated cells (**Figure 5A**), a clear cell damage was observed in three dimensional analysis of treated cells (**Figure 5B**). The height of untreated cells was found to be 1699 nm (**Figure 5C**), whereas the treated cells showed 15 nm height (**Figure 5D**) confirming the rupture and loss of rigidity in the cells. The roughness of treated and untreated bacterial cells was measured as the root mean square (rms) values. As indicated in **Figures 5C,D**, the treated cells showed lower rms value (2.17 nm) than untreated cells (116.19 nm). Overall, the SEM (showing morphological changes), TEM (showing ultrastructure cell damages), and AFM (showing three dimensional morphological changes) together substantiate the AgNps induced bacterial cell distortion and disintegration.

## Discussion

The synthesis of AgNps through *J. curcas* leaf extract exhibited a time dependent color change correlated with the peaks obtained with UV-vis spectroscopy. The main advantage of using plants for AgNp synthesis is that they provide the combination of natural capping agents (such as amino acids, enzymes, polysaccharides, terpenoids, alkanoids, phenolics, and vitamins, etc.) for stabilization of AgNps in suspension (Ahmed et al., 2016). Recent research has reported the presence of many bioactive compounds in the aqueous/solvent leaf extracts of *J. curcas* responsible for synthesis, stabilization/capping of Jc-AgNps and also for its efficient antimicrobial activity (Kalimuthu et al., 2010; Ahirrao et al., 2011; Najda et al., 2013; Chauhan et al., 2015). The vibrational bands (FTIR) observed in the Jc-AgNps indicated the presence of various compounds (free amines, alkanoids, and flavonoids), which were earlier reported for synthesis and stabilization of nanoparticles (Gole et al., 2001; Lee et al., 2014; Joseph and Mathew, 2015).

The value of zeta potential is zero at iso-electric point proving its least stability, whereas the highly negative and positive zeta potential are considered to be more stable (Sadeghi and Gholamhoseinpoor, 2015) validating the high stability (-23.4 mV) of synthesized Jc-AgNps in colloidal suspension. The spherical and irregular shaped nanoparticles of varied sizes could be attributed to difference in their formation timings as suggested in various studies (Kasthuri et al., 2009; Sadhasivam et al., 2010). Rajasekharreddy et al. (2010) have also synthesized AgNps from aqueous *J. curcas* leaf extract and reported the average particle size of 20 nm. The present finding showed the average particle size of 43 nm (DLS) which is very close with the above. However, more uniformity and smaller size in previous report can arise from controlled reaction rate and higher temperature conditions induced by incubation in sunlight as opposed to dark synthesis in our study. AFM more clearly depicts the agglomeration and actual size of nanoparticles. Previously, Hemath Naveen et al. (2010) used AFM to depict agglomeration and irregular surfaces of biologically formed nanoparticle. In contrast, the surface roughness properties (rms: 1.31) analyzed using AFM in current study showed smooth nanoparticles without any cracks.

The trend of inhibitory zone produced by Jc-AgNps showed that the Gram-negative bacteria were more sensitive than Gram-positive bacterial strains. The highest ZOI was obtained with *E. coli* at 30 μl (0.5 mg/ml) followed by *P. aeruginosa*, making them more sensitive than the other bacteria tested in this study. Birla et al. (2009) reported that green synthesized AgNps enhanced the antimicrobial activity of antibiotics against *E. coli* and *P. aeruginosa*. Similarly, Sondi and Salopek-Sondi (2004) have observed 70% growth inhibition of *E. coli* cells at 10 μg/cm^3^ of silver nanoparticle. In another study, Awwad et al. (2012) have described the efficient antibacterial activity of *Olea europaea* leaf synthesized AgNps, suggesting *L. monocytogenes* (ZOI: 20 mm) to be more sensitive than *S. aureus* (ZOI: 10 mm) as displayed in the present study. Similar pattern for antibacterial potential of Jc-AgNps was observed using the microdilution method, where the MIC and MBC values for Gram-positive bacteria were higher than that for Gram-negative bacteria. Studies have pointed that thicker cell wall of Gram-positive bacteria protects the penetration of silver ions into cytoplasm, therefore the effect of AgNps is more pronounced in Gram-negative bacteria than Gram-positive bacteria (Feng et al., 2000; Shrivastava et al., 2007). This was further confirmed in the present study as the cells of *S. enterica* showed significant cell damage even at ½ MIC level of Jc-AgNps, and at the MIC level only ghost like cell remains were observed under SEM examination. Although Jc-AgNps treated *L. monocytogenes* cells were observed to be deformed and shrunk, the damage was less pronounced than that in *S. enterica* cells.

The more comprehensible cell damage and clear penetration and interaction of silver nanoparticle with cell membrane was observed under TEM micrograph. The 48 h Jc-AgNps treated cells of *L. monocytogenes* showed complete cell distortion with visible Jc-AgNps surrounding damaged cell (**Figure 4B**). Reports have pointed the similar trend of interaction, penetration and damage of bacterial cells by AgNps (Xu et al., 2004; Raffi et al., 2008). Some previous studies (Tyagi and Malik, 2010) have assessed the AFM analysis to show distinctive change in cell surface and structure. It was observed that a remarkable decrease occurs in height and root mean square (pertaining to surface roughness) values of treated cells which is correlated with the observations of three dimensional modification in *L. monocytogenes* cells due to Jc-AgNps treatments in the present study. Literature reports have suggested that the efficient activity of AgNps is due to its large surface area to attach with microorganisms (Umadevi et al., 2011; Ahmed et al., 2016). AgNps were believed to interact with sulfur-containing proteins of membrane and also with phosphorous containing DNA of bacterial cell, thereby inhibiting cell division leading to cell death (Feng et al., 2000; Rai et al., 2009).

The antimicrobial activity of leaf (Kalimuthu et al., 2010), stem bark (Igbinosa et al., 2009), and roots (Arekemase et al., 2011) of *J. curcas* have been reported in various studies, but very few reports have used *J. curcas* for the synthesis of AgNps (Bar et al., 2009a,b; Rajasekharreddy et al., 2010). None of these reports have tested the antibacterial potential and interaction of synthesized Jc-AgNps with bacterial cells. Hence, the present study is relevant, as this is the first systematic report on antibacterial potential of Jc-AgNps against food borne microbes. Results can be useful for designing ways to control food borne pathogens. Some reports have exploited the potential of AgNps for food protection by preparing AgNps loaded food packaging materials with efficient antibacterial activity against *E. coli* and *S. aureus* (Tankhiwale and Bajpai, 2009; De Moura et al., 2012). However, these studies have employed chemical/physical methods for synthesizing AgNps which are quite expensive and potentially hazardous, especially for food application (Ahmed et al., 2016). Further research to use such green synthesized and stable AgNps for designing materials for varied applications is warranted.

## Conclusion

The present study demonstrated significant antibacterial activity of *J. curcas* leaf extract (aqueous) synthesized silver nanoparticles (Jc-AgNps). The analytical techniques such as UV-vis, Zeta potential, SEM-EDX, XRD established the formation of stable crystalline AgNps, whereas spherical and irregular shape of Jc-AgNps (20–100 nm) was confirmed by AFM, SEM, TEM, and DLS analysis. The FTIR analysis gave strong indications about the involvement of various biological compounds responsible for formation and stabilization of Jc-AgNps in suspension. The synthesized Jc-AgNps showed significant bactericidal activity against Gram-positive and Gram-negative bacteria and were highly effective against *E. coli*. A detailed microscopic examination of *L. monocytogenes* using SEM and AFM showed a visible morphological distortion, whereas TEM clearly spotlighted the impact of Jc-AgNps on cell’s ultrastructure, showing membrane pits, intracellular Jc-AgNps accumulation and cell damage in treated cells. The antibacterial potential of Jc-AgNps can be useful in designing need based applications in various sectors (Environment, healthcare, textiles, etc) i.e., water purification, nano-silver based antimicrobial paints/coatings, silver dressings for wound management, AgNp impregnated clothings which restricts the growth of odor causing bacteria.

## Author Contributions

NC, AT, and PK designed the experiments and performed the experiments. AT and AM kept eyes on data analysis and did the manuscript compilation. All authors reviewed the manuscript before submission.

## Conflict of Interest Statement

The authors declare that the research was conducted in the absence of any commercial or financial relationships that could be construed as a potential conflict of interest.

## Supplementary material

FIGURE S1 **(A)** Three different tubes (1, 2, 3) monitored at 24, 48, and 72 h (Tube 1) AgNO_3_ solution (0.002 M), (Tube 2) Plant aqueous extract (*Jatropha curcas* leaves), (Tube 3) Synthesis of Jc-AgNps (plant extract with 0.002 M AgNO3 solution in 1:10 ratio). **(B)** UV-vis absorption spectra of Jc-AgNPs synthesized at room temperature by treating 0.002 M AgNO_3_ with *J. curcas* leaf extract at different time intervals (24, 48, and 72 h).

FIGURE S2 **(A)** Calibration curve of silver at different concentrations (2, 6, 8 ppm); **(B)** Quantification of silver in the sample (Jc-AgNp) by Microwave plasma-atomic emission spectrometry (MP-AES).

FIGURE S3 **Physical characterization of green synthesized Jc-AgNps. (A)** Size distribution by intensity dynamic light scattering (DLS); Z average 43.67 nm (diam.); **(B)** Zeta potential (-23.4 mV) distribution, **(C)** X-ray diffraction (XRD) patterns of Jc-AgNPs powder, **(D)** FTIR spectrum of vacuum dried powder of Jc-AgNPs.

FIGURE S4 **Scanning electron microscopic analysis (SEM), (A)** SEM micrograph of untreated *Salmonella enterica* cells showing normal and well organized structure, (B) SEM micrograph of Jc-AgNps treated (½ MIC) *S. enterica* cells showing deformation and aggregation, (C) SEM micrograph of Jc-AgNps treated (MIC) *S. enterica* cells showing extremely damaged remains.
