# Supplementary figures and images for: Antibacterial Potential of Jatropha curcas Synthesized Silver Nanoparticles against Food Borne Pathogens
Source: Front Microbiol. 2016 Nov 8;7:1748. doi: 10.3389/fmicb.2016.01748 (PMC5099242; doi:10.3389/fmicb.2016.01748)

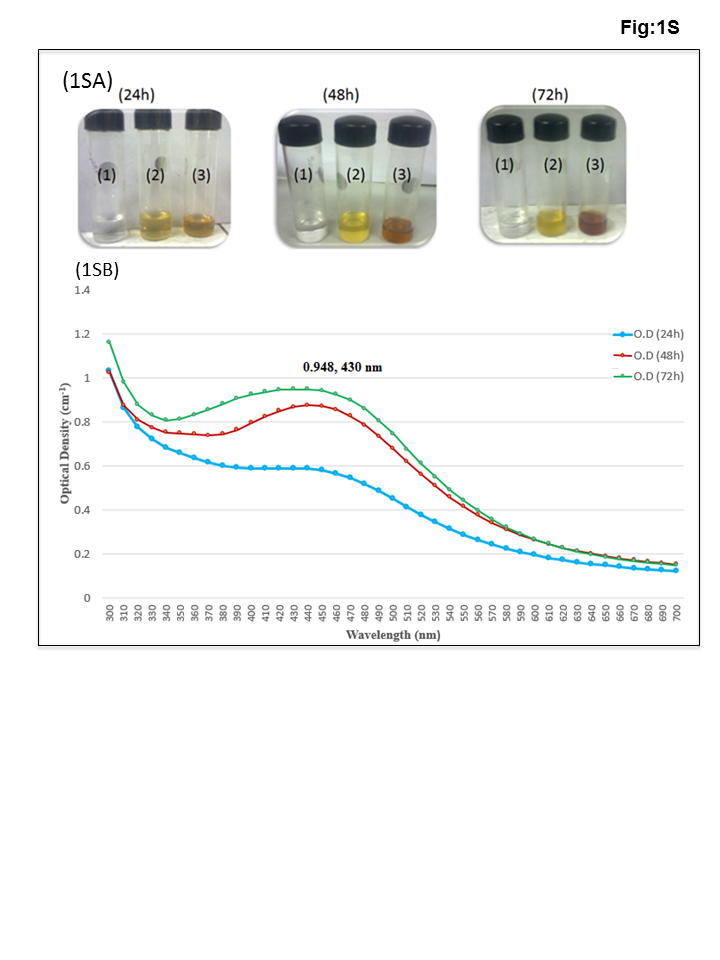

Supplement: FIGURE S1 — (A) Three different tubes (1, 2, 3) monitored at 24, 48, and 72 h (Tube 1) AgNO3 solution (0.002 M), (Tube 2) Plant aqueous extract (Jatropha curcas leaves), (Tube 3) Synthesis of Jc-AgNps (plant extract with 0.002 M AgNO3 solution in 1:10 ratio). (B) UV-vis absorption spectra of Jc-AgNPs synthesized at room temperature by treating 0.002 M AgNO3 with J. curcas leaf extract at different time intervals (24, 48, and 72 h). [file Image_1.TIF]

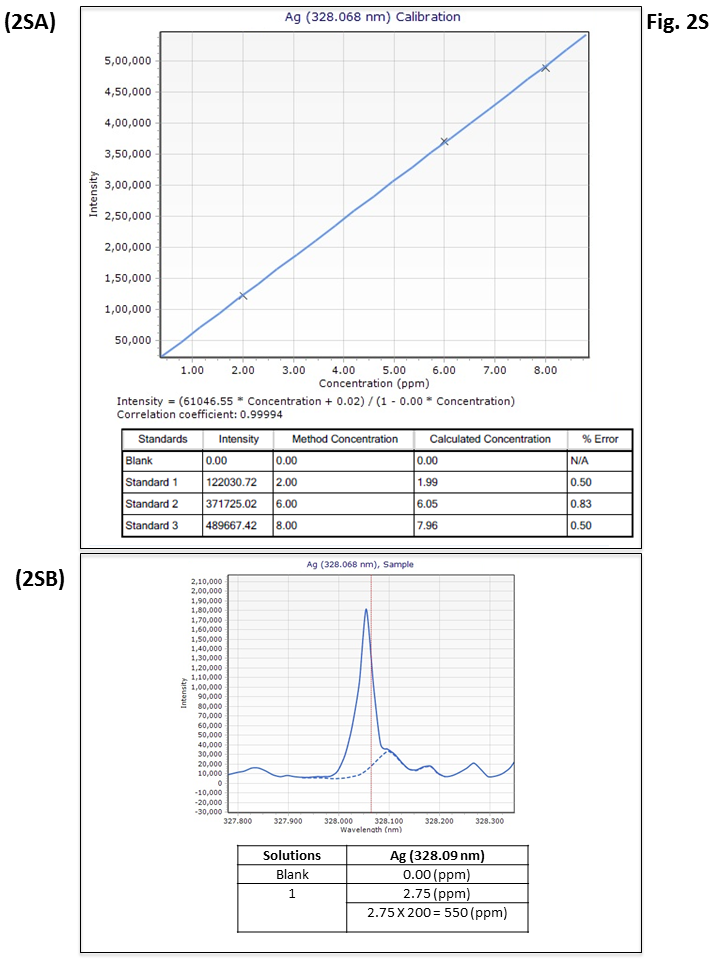

Supplement: FIGURE S2 — (A) Calibration curve of silver at different concentrations (2, 6, 8 ppm); (B) Quantification of silver in the sample (Jc-AgNp) by Microwave plasma-atomic emission spectrometry (MP-AES). [file Image_2.TIF]

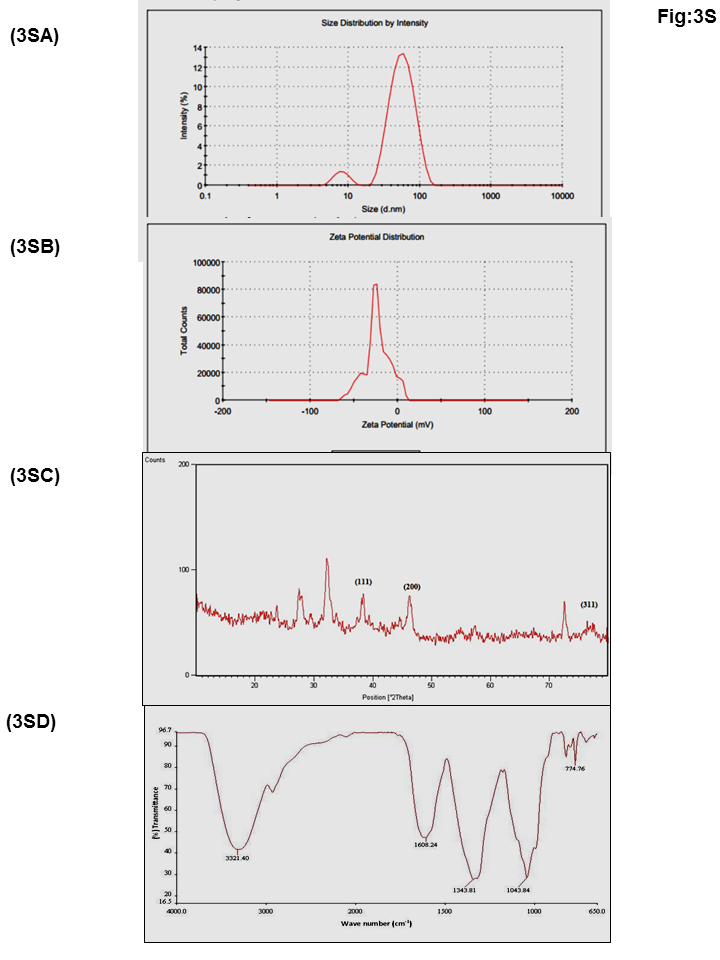

Supplement: FIGURE S3 — Physical characterization of green synthesized Jc-AgNps. (A) Size distribution by intensity dynamic light scattering (DLS); Z average 43.67 nm (diam.); (B) Zeta potential (-23.4 mV) distribution, (C) X-ray diffraction (XRD) patterns of Jc-AgNPs powder, (D) FTIR spectrum of vacuum dried powder of Jc-AgNPs. [file Image_3.TIF]

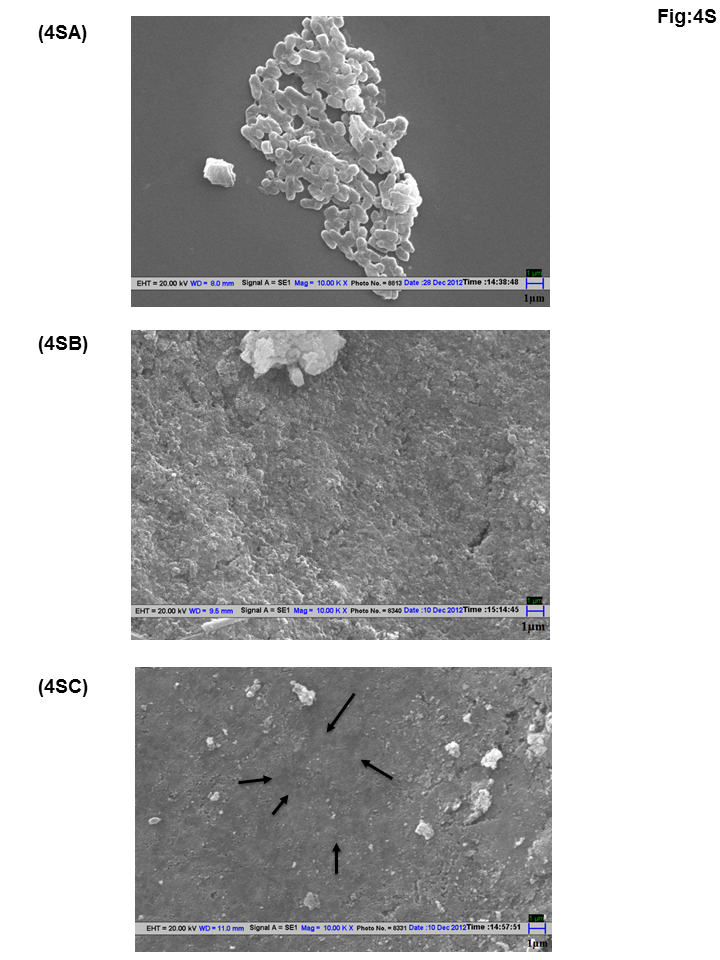

Supplement: FIGURE S4 — Scanning electron microscopic analysis (SEM), (A) SEM micrograph of untreated Salmonella enterica cells showing normal and well organized structure, (B) SEM micrograph of Jc-AgNps treated (½ MIC) S. enterica cells showing deformation and aggregation, (C) SEM micrograph of Jc-AgNps treated (MIC) S. enterica cells showing extremely damaged remains. [file Image_4.TIF]
